# Modulation of Intestinal Microbiota in *Solea senegalensis* Fed Low Dietary Level of *Ulva ohnoi*

**DOI:** 10.3389/fmicb.2019.00171

**Published:** 2019-02-07

**Authors:** Silvana T. Tapia-Paniagua, Milena Fumanal, Victoria Anguís, Catalina Fernández-Díaz, F. Javier Alarcón, Miguel A. Moriñigo, M. Carmen Balebona

**Affiliations:** ^1^Departamento de Microbiología, Facultad de Ciencias, Universidad de Málaga, Málaga, Spain; ^2^IFAPA Centro El Toruño, El Puerto de Santa María, Spain; ^3^Departamento de Biología y Geología, Universidad de Almería, Almería, Spain

**Keywords:** microbiome, *Ulva*, algae, high-throughput sequencing, *Solea senegalensis*

## Abstract

Gastrointestinal (GI) microbiota has a relevant role in animal nutrition, modulation of the immune system and protection against pathogen invasion. Interest in algae as source of nutrients and functional ingredients for aquafeeds is increasing in order to substitute conventional feedstuffs by more sustainable resources. The diet is an important factor in the modulation of the microbiota composition, and functional ingredients have been proposed to shape the microbiota and contribute benefits to the host. However, fish microbiome research is still limited compared to other hosts. *Solea senegalensis* is a flat fish with high potential for aquaculture in South Europe. In this study, a characterization of the microbiome of *S. senegalensis* (GI) tract and the effects of feeding *Ulva ohnoi* supplemented diet has been carried out. Differences in the composition of the microbiota of anterior and posterior sections of *S. senegalensis* GI tract have been observed, *Pseudomonas* being more abundant in the anterior sections and *Mycoplasmataceae* the dominant taxa in the posterior GI tract sections. In addition, modulation of the GI microbiota of juvenile Senegalese sole fed for 45 days a diet containing low percentage of *U. ohnoi* has been observed in the present study. Microbiota of the anterior regions of the intestinal tract was mainly modulated, with higher abundance of *Vibrio* spp. in the GI tract of fish fed dietary *U. ohnoi*.

## Introduction

Fish farming is a growing industry that represents an alternative to fishing. Senegalese sole (*Solea senegalensis*) is a flatfish with great potential for marine aquaculture due to its high market value and consumer demand. Recent management and technical improvements in *S. senegalensis* culture are leading to important progress in productivity, but there are still unsolved questions regarding nutrition of this species under cultured conditions (Morais et al., 2016).

In recent years, algae are being considered as potential sources of dietary ingredients for aquafeeds. Among them, *Ulva* species are a good source of protein, minerals and vitamins, especially vitamin C (Ortiz et al., 2006; Garcia-Casal et al., 2007). In addition, when added to fish meal at low percentages, benefits such as improved growth, feed efficiency, nutrient utilization, and disease resistance have been described in several fish species (Mustafa and Nakagawa, 1995; Wassef et al., 2005; Valente et al., 2006; Ergün et al., 2008; Moutinho et al., 2018). In this way, it has been reported that 5% dietary *Ulva* can be used as functional ingredient for feeding fish, whilst higher supplementation levels may compromise fish growth (Valente et al., 2006; Moroney et al., 2017). For this reason, experimental feeds for *S. senegalensis* were supplemented with 5% *Ulva ohnoi* in the present work.

It is well established that the gastrointestinal (GI) microbiota has a relevant role in animal nutrition, development and resistance against pathogens (Llewellyn et al., 2014). In the case of fish, several studies have reported the role of GI microbiota in nutrition, epithelial renewal, modulation of immune system and protection from pathogen invasion (Gatesoupe et al., 1997; Rawls et al., 2004; Bates et al., 2006; Salinas et al., 2006; Semova et al., 2012; Ingerslev et al., 2014a,b; Wang et al., 2018). Regarding nutrition, GI microbiota is comprised of a range of microorganisms capable to produce vitamins enzymes such as lipases, amylases, proteases, chitinases and phytases, which contribute to the host digestion through hydrolysis of aquafeed components (Ray et al., 2012). In addition, GI microbiota participate in epithelial renewal and enhancement of β-catenin stability in enterocytes (Cheesman et al., 2011). Fish GI microbiota is a key component in the development, maturation and modulation of the immune system (Bates et al., 2006). Microbiota and microbiota derived products influence both systemic and mucosal immune response through microbe-associated molecular patterns (MAMPs) interaction with pattern recognition receptors (PRRs) in host cells. Teleost gut-associated lymphoid tissues (GALT) have significant numbers of antigen-presenting cells that participate in the initial stages of the immune response. A role in the modulation of cytokine and immunoglobulin levels as well as activation of cellular response including B and T cells, has been reported for fish GI microbiota (Kelly and Salinas, 2017).

Microbiota composition is shaped by different factors including host species, trophic level, environment, feeding habits (Romero et al., 2014; Eichmiller et al., 2016; Goncalves and Gallardo-Escárate, 2017), probiotics (Merrifield et al., 2010) and functional ingredients or nutraceuticals (Ringø et al., 2006, 2016). Also, variations in physiological parameters along the GI tract have been demonstrated to shape microbial communities (Ye et al., 2014). However, despite some information is already available, fish microbiome research is still limited compared to other hosts.

At present, as in other fish species, inclusion of macroalgae in aquafeeds is being considered for *S. senegalensis*. However, this species presents important particularities compared to other marine aquaculture species, especially regarding the GI tract (Morais et al., 2016) and, as far as we know, no information about the potential effects of dietary incorporation of *Ulva* on the GI microbiota is available.

Our current information on the gut microbiota of *S. senegalensis* is in part derived from analysis of culture dependent techniques (Martin-Antonio et al., 2007). However, percentages of culturable bacteria in fish GI tract are under 1% (Romero and Navarrete, 2006; Navarrete et al., 2009; Aguilera et al., 2013; Romero et al., 2014). Clone libraries based on 16S rDNA and denaturing gradient gel electrophoresis (DGGE) (Tapia-Paniagua et al., 2010) have also been applied to the study of *S. senegalensis* GI microbiota, especially to evaluate the effect of probiotic supplementation of the diet (Tapia-Paniagua et al., 2014a). In contrast to previous methodologies, Next Generation Sequencing (NGS) of 16S rRNA genes allows unbiased identification of rare, as well as abundant, bacterial members of the gut microbiota. Ghanbari et al. (2015) highlighted the potential of NGS platforms for the analysis of fish gut microbial ecology and its capacity to improve the knowledge of the microbial community profiles of fish GI microbiota. Still, few works have addressed the understanding of the composition and structure of fish GI microbial populations with high throughput sequencing and no studies are available on GI microbiota of *S. senegalensis*. The knowledge on fish intestinal microbiota will facilitate the development of effective strategies for manipulating GI microbial communities to promote fish health and productivity.

In this study, a characterization of the microbiome of *S. senegalensis* GI tract was carried out based on 16S rRNA amplicon sequencing (V3-V4 region) using the Illumina’s MiSeq platform in order to better understanding the modulation of the microbiota by the supplementation of *U. ohnoi* in fish diet. Differences in the microbiota composition between the anterior and posterior sections and the effect of the presence of *Ulva* in the diet are described in the present study.

## Materials and Methods

### Diet Composition and Preparation

*Ulva ohnoi* Hiraoka and Shimada strain UOHN120810 was isolated from the outlet channel of fish cultivation facilities in IFAPA El Toruño (El Puerto de Santa María, Cádiz, Spain) and maintained in culture. To obtain the biomass needed for the trial, stock cultures were up-scaled to 1000 L tanks. *Ulva* biomass (1 kg m^-3^) was cultured for 2 weeks under natural photoperiod light using a modified f/2 medium (Guillard, 1975) with 1.8 mM nitrate and 0.1 mM phosphate prepared with filtered (0.2 μm) natural seawater. Algae were harvested, rinsed with tap water, freeze-dried and kept in a dry place until used as ingredient in the experimental diet.

### Experimental Diets

Two isonitrogenous (55% on dry weight basis) and isolipidic (15% on dry weight) experimental diets were manufactured at the CEIA3-Universidad de Almería facilities (Service of Experimental Diets)^1^. *Ulva* diet was formulated to include 5% (w/w) dry *U. ohnoi* biomass. An algae-free diet was used as control. The ingredient composition of experimental diets is shown in Table 1.

**Table 1:** Ingredient composition of the experimental diets used in the feeding trial.

	Control diet	*Ulva* diet
*Ingredients* (g kg^-1^ DM)		
Fishmeal LT^1^	674	660
*Ulva meal*		50
Squid meal^2^	50	50
Fish protein hydrolysate^3^	50	50
Krill meal^2^	10	10
Shrimp meal^2^	10	10
Gluten meal^2^	20	20
Soybean protein concentrate^4^	20	20
Fish oil	29	28
Soybean lecithin	20	20
Maltodextrin	46	11
Choline chloride^5^	10	10
Vitamin and mineral premix^6^	20	30
Guan gum^2^	15	15
Alginate^2^	15	15

*^*1*^(69.4% crude protein, 12.3% crude lipid), Norsildmel (Bergen, Norway); ^*2*^Local provider (Lifebioencapsulation SL, Almería, Spain); ^*3*^(81% crude protein, 8.8% crude lipid) Sopropeche (France); ^*4*^(65% crude protein, 8% crude lipid) DSM (France); ^*5*^Sigma-Aldrich (Madrid, Spain); ^*6*^Mineral and vitamin premix according to Vizcaíno et al. (2018). Proximate composition of control diet: 55.2% crude protein, 12.4% crude lipid, 3.0% fiber, 12.8% ash), and *Ulva* diet: 55.0% crude protein, 12.0% crude lipid, 3.3% fiber, 13.1% ash.*

Feed ingredients were finely ground and mixed in a vertical helix ribbon mixer (Sammic BM-10, 10-L capacity, Sammic, Azpeitia, Spain) before fish oil and diluted choline chloride were added. All the ingredients were mixed together for 15 min, and then water (300 mL kg^-1^) was added to the mixture to obtain homogeneous dough. The dough was passed through a single screw laboratory extruder (Miltenz 51SP, JSConwell Ltd., New Zealand), to form 1–2 mm (diameter) and 2–3 mm (length) pellets. The extruder barrel consisted of four sections and the temperature profile in each section (from inlet to outlet) was 100C, 95, 90, and 85°C, respectively. Finally, pellets were dried at room temperature for 24 h and kept in sealed plastic bags at -20°C until use.

### Fish Maintenance and Sampling Procedures

Juvenile Senegalese sole (*Solea senegalensis*) with mean initial body weight of 10.7 ± 2.9 g were obtained from a commercial hatchery (Cupimar S.A., San Fernando, Cádiz, Spain) and transported to the research facilities of the IFAPA El Toruño (El Puerto de Santa María, Cádiz, Spain). Fish were stocked at 1.5 kg m^-2^ in six tanks connected to a closed recirculation consisting of a mechanical filter, a skimmer, ultraviolet light and a biofilter. Fish were fed daily at 2% fish biomass with an experimental diet considered as control diet for 10 days for acclimatizing the fish to the experimental conditions. After the acclimation period, fish (12.3 ± 2.0 g mean body weight) of each set of three tanks were fed with two different experimental diets: control diet (control diet) and diet containing *U. ohnoi* 5% (*Ulva* diet) for 45 days at a rate of 3% of their body weight. Different parameters were registered during all the experimental period. The temperature, pH, salinity and oxygen were maintained constant at 19.9 ± 0.7°C, 7.8 ± 0.2; 25.7 ± 1.5‰ and 7.0 ± 0.4 mg L^-1^ respectively. Nitrite and ammonia were checked once a week (values were always below 0.1 mg L^-1^).

At the end of the feeding trial (45 days), fish were fasted for 24 h for sampling. Soles (*N* = 6) were carefully taken from their respective tanks and transferred to a new tank containing clove oil (200 ppm) to euthanize. Whole intestines of six fish per treatment (2 per tank) were aseptically removed, divided into two equal length sections and stored separately in Trisure, -80°C, until further analysis.

### DNA Extraction, PCR Amplification and Sequencing

Individual intestinal content was collected with 1 mL PBS pH 7.2, and 1 mL aliquot per sample was centrifuged (1000 × *g*, 5 min). Total DNA was extracted from each sample (*N* = 12) according to manufacturer specifications using Trisure (Bioline, Spain) and purified by sodium acetate precipitation (20 μl DNA was precipitated with 2 μl sodium acetate 3 M and 46 μl isopropanol). Then, DNA was centrifuged for 3 min at 12,000 × g, 4°C. Supernatants were discarded and the pellets rinsed with cold 70% ethanol and centrifuged for 5–15 min at 12,000 × *g*, 4°C. Supernatants were discarded again and pellets air dried. Finally, DNA was resuspended in water. DNA quality and integrity was visualized by electrophoresis in 1% agarose gels, stained with GelRed Nucleic Acid Stain 20000x (InTRON Biotechnology, Seoul, Korea). Concentration and purity were determined by using Qubit 2.0 fluorometer (Thermo Fisher Scientific, Germany). DNA was stored at -20°C for further processing and 30 ng were used for subsequent analyses.

Libraries were constructed by Chunlab, Inc., (Seoul, South Korea) using the Illumina MiSeq Platform. Briefly, Illumina paired-end sequencing of each sample was carried out by using the primers 341F CCTACGGGNGGCWGCAG and 805R GACTACHVGGGTATCTAATCC (ChunLab), targeting V3-V4 regions of 16 S rRNA gene.

After removing Illumina barcodes and demultiplexing, paired-end reads were merged by using PANDAseq software (Masella et al., 2012) and processed for primer sequence trimming according to BIOiPLUG in-house pipeline. Then, the assembled reads were quality-filtered one sample at a time using BIOiPLUG pipeline, excluding reads < 80 bp or >2,000 bp long, with Phred quality score average below 25. Non-specific PCR amplicons not predicted as a 16 S gene by the Hidden Markov Model (HMM) based search were also removed. Singleton sequences were excluded in the subsequent analyses. Following, denoising was performed with DUDE.Seq software and sequences were de-replicated.

UCHIME (Edgar et al., 2011) was used to detect and remove chimera against BIOiPLUG’s chimera-free reference database. The remaining representative, non-chimeric sequences were then subjected to taxonomic assignment against the EzBioCloud 16S database (Yoon et al., 2017), with 97% 16S similarity as the cutoff and clustered into operational taxonomic units (OTUs) using UCLUST open reference method (Rideout et al., 2014).

After generating the taxonomic profile of microbiome samples, comparison of taxa present in the samples was carried out. Random subsampling was conducted to normalize the data size to 15,000 reads, because the total number of reads that remained after pre-processing varied depending on the samples and this size was below the minimum number of reads obtained in all the samples. All statistical analyses were performed using this subset. To determine the level of sequencing depth, rarefaction curves were obtained by plotting the number of observed OTUs against the number of sequences and Good’s coverage coefficient calculated. Alpha diversity was estimated based in Shannon-Wiener, Chao1 and Simpson indexes, in order to assay taxonomic and phylogenetic structure diversity, respectively. The results are generally presented at phylum and genus taxonomic level.

### Calculations and Statistical Analysis

Growth parameters and survival rate were calculated according to the following expressions:

Weight gain rate (WGR)(%) = 100 × (final body weight – initial body weight)/initial body weight; Feed conversion ratio (FCR) = Dry feed consumed (g)/wet weight gain (g) and Survival rate (%) = 100 × final fish number/initial fish number.

Good’s coverage, rarefaction curves and alpha diversity indices including CHAO1 richness estimation and community diversity (Shannon index and Simpson) were calculated according to Caporaso et al. (2011) using ChunLab platform.

Normality (Shapiro–Wilk) and homogeneity of variance (Levene test) were checked and statistical significance of growth data and alpha diversity values was determined by One-Way Analysis of Variance (ANOVA) followed by Tukey test to determine significance of paired comparisons. All tests were performed with XLSTAT software.

Multivariate analysis of OTU data was performed via Principal Coordinate Analysis (PCoA) of OTU profiles using Bray Curtis metric in order to depict differences between microbiota of each group. In addition, to test the hypothesis of no differences between the microbiota of the GI sections and diets assayed, dissimilarity matrices obtained with Bray-Curtis index were analyzed by Permutation multivariate analysis of variance (PERMANOVA) with 999 permutations by using PAST software (Hammer, 2001) version 3.16. Linear discriminant analysis (LDA) effect size (LEfSe) (Segata et al., 2011) was used to characterize microbial differences of biological relevance between the diets within the two different GI sections and between sections within the same diet. The LEfSe analysis was performed using an alpha value of 0.05 for both the factorial Kruskal-Wallis rank sum test and pairwise Wilcoxon test and a threshold of 2.0 for the LDA. BIOiPLUG was used to carry out LefSe analysis.

### Ethics Statement

All studies involving fish were conducted in strict accordance with Guidelines established by the European Union (2010/63/UE) and the Spanish legislation (RD 1201/2005 and RD 53/2013) for the use of laboratory animals. All procedures were authorized by the Bioethics and Animal Welfare Committee of the Institute of Agricultural and Fisheries Research and Training (IFAPA), and given the registration number 17/11/2016/171 according to the national authorities for regulation of animal care and experimentation.

## Results

### Fish Growth, Sequencing Overview and Microbiota Characterization

No fish mortality occurred during the experimental period; however, slightly decreased final body weight and weight gain rate values were obtained for fish fed with *U. ohnoi* supplemented diet (Table 2).

**Table 2:** Growth performance and survival of juvenile *Solea senegalensis* specimens fed control (Control) and *Ulva ohnoi* (5%) supplemented (*Ulva*) diets for 45 days.

	FBW(g)	WGR (%)	FCR	Survival (%)
Control diet	28.22 ± 6.12^a^	129.6 ± 40.8^a^	0.85 ± 0.02^a^	100
*Ulva* diet	24.88 ± 4.18^b^	101.7 ± 38.9^b^	1.06 ± 0.01^b^	100

*FBW, final body weight; WGR, weight gain rate; FCR, feed conversion ratio. Values are average ± SD. Different letters indicate significant differences between the experimental diets (*p* < 0.05).*

DNA was extracted from 24 samples, six *S. senegalensis* specimens per diet and 2 intestinal regions per fish specimen. In total, 653,198 raw reads were obtained for both forward and reverse directions after sequencing. The mean read depth per sample was 27,216.58 ± 7,578 (mean ± SD) sequences per read direction. After removing non-specific amplicons, amplicons not assigned to the target taxon, and chimeras in the initial quality filtering, 613,800 sequences passed with a mean of 25,575.00 ± 6,677.07 (mean ± SD) sequences per sample. Singletons were removed and a total of 3638 OTUs at a 97% gene similarity cut off against EzBioCloud database were used for subsequent analysis. Random subsampling was conducted to normalize the data size to 15,000 reads.

### Alpha Diversity

Alpha diversity indices were calculated for microbiota data of both control and *Ulva* diets (Table 3).

**Table 3:** Alpha diversity of bacterial communities in the anterior (A) and posterior (P) intestinal tract regions of juvenile *Solea senegalensis* specimens fed control (Control) and *Ulva ohnoi* (5%) supplemented (*Ulva*) diets for 45 days.

Diet	OTU	Chao1	Shannon	Simpson	Good’s coverage (%)
Control A	205.67 ± 48.42	230.53 ± 58.50	1.01 ± 0.15^∗^= 1	0.69 ± 0.07^∗^= 1	99.7 ± 0.1
Ulva A	201.50 ± 47.48	229.40 ± 41.30	1.60 ± 0.54^∗^	0.48 ± 0.19^∗^	99.7 ± 0.1
Control P	213.33 ± 53.63	227.42 ± 62.71	1.72 ± 0.40= 1	0.39 ± 0.14= 1	99.7 ± 0.2
*Ulva* P	271.33 ± 88.41	280.67 ± 81.98	2.23 ± 0.62	0.30 ± 0.14	99.8 ± 0.1

*Values are average ± SD. ^∗^Significant differences between the experimental diets for the same gastrointestinal region; = 1significant differences between GI sections in fish fed the same diet, *p* < 0.05.*

Mean Good’s coverage estimator value was 99.7 ± 0.1 (mean ± SD) (ranging from 99.7 to 99.8%), indicating adequate sequencing depth. No significant differences in species richness were observed in the microbiota of the samples studied. Thus, no statistically significant differences in OTU number or Chao1 richness estimates were detected between control and *Ulva* diet microbiota neither in anterior or posterior GI sections. However, both Shannon and Simpson indices showed statistically significant differences in the anterior portion of the GI tract based on the diet received by the fish (*p* = 0.028 and *p* = 0.027, respectively), higher diversity being observed in fish fed *Ulva* diet. On the contrary, no significant changes in Shannon and Simpson indices were detected between the microbiota of control and alga supplemented groups in the posterior GI sections (*p* > 0.05).

When anterior and posterior sections of the GI tract were compared, higher Shannon diversity and lower Simpson index values were detected in the microbiota of the posterior GI region of fish receiving the diet devoid of *Ulva* (*p* = 0.004 and *p* = 0.001, respectively), with no significant differences in diversity in the case of fish fed *Ulva* supplemented diet (*p* > 0.05).

### *S. senegalensis* GI Microbiota Composition

**FIGURE 1:**
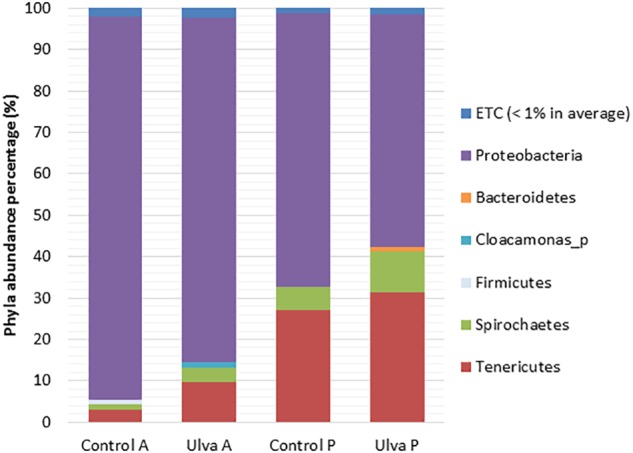
Gut microbiota composition (relative OTU composition) at phylum level of the gastrointestinal tract of *S. senegalensis* specimens fed control (Control) and *Ulva ohnoi* supplemented *(Ulva)* diet for 45 days. A, anterior half region; P, posterior half region.

Phyla detected with average values above 1% in samples from fish fed with control and *Ulva* supplemented diets included Proteobacteria, Spirochaetes, and Tenericutes in both GI sections, whilst Bacteroidetes was only detected in the posterior sections of fish fed with *Ulva* diet (Figure 1). Proteobacteria was the dominant phylum in all the cases, higher percentages being found in the microbiota of the anterior GI region of fish fed with control and *Ulva* supplemented diets (92.62 and 83.32%, respectively) compared to the posterior regions (66.11 and 56.17%, respectively). Interestingly, GI microbiota of fish fed with *Ulva* diet contained lower Proteobacteria levels compared to the fish fed control diet. High variability in relative abundance values was observed in the microbiota of fish fed with *Ulva* diet, especially in the anterior sections. Nevertheless, it was possible to observe overrepresentation of Tenericutes members in the posterior GI sections, especially in fish fed *Ulva* diet. Other phyla such as Actinobacteria and Firmicutes were detected with average values below 1% in the microbiota of all samples analyzed, although Firmicutes abundance reached average values above 1% (1.06%) only in the anterior GI sections of control fish. Finally, Cloacamonas_p was detected in the microbiota of the anterior sections in *Ulva* fed fish (1.15%).

**FIGURE 2:**
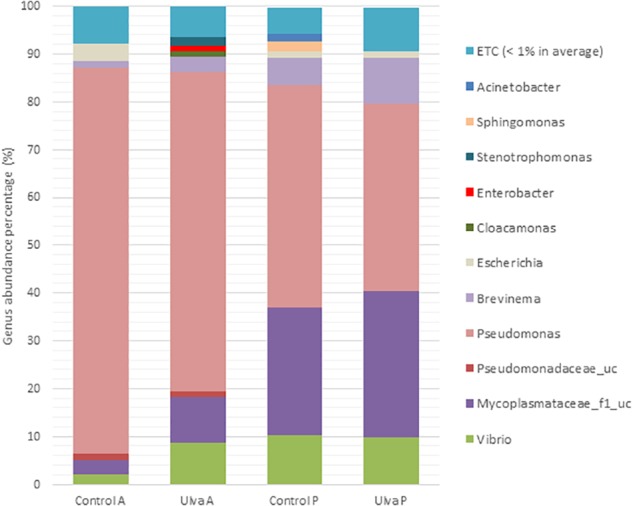
Gut microbiota composition (relative OTU composition) at genus level of the gastrointestinal tract of *S. senegalensis* specimens fed control (Control) and Ulva ohnoi (*Ulva*) supplemented diets for 45 days. A, anterior half region; P, posterior half region.

When composition of the microbiota was considered at genus level, *Pseudomonas* was the most abundant taxon in the anterior GI region regardless of the diet received by *S. senegalensis* specimens (80.87 and 66.74% in control and *Ulva* group, respectively) (Figure 2). These percentages decreased in the posterior sections, the lowest percentages corresponding to fish fed with *Ulva* diet (39.21%,). On the contrary, abundance percentages of Mycoplasmataceae_f1_uc and *Brevinema* increased in the posterior regions, this latter especially in *Ulva* diet fed specimens. Members of *Vibrio* genus were detected in the microbiota of all GI regions regardless of the diet. However, they were overrepresented in the anterior region of fish fed *Ulva* diet (8.67%) compared to those fed control diet (2.03%) (Figure 2).

### Beta Diversity

**FIGURE 3:**
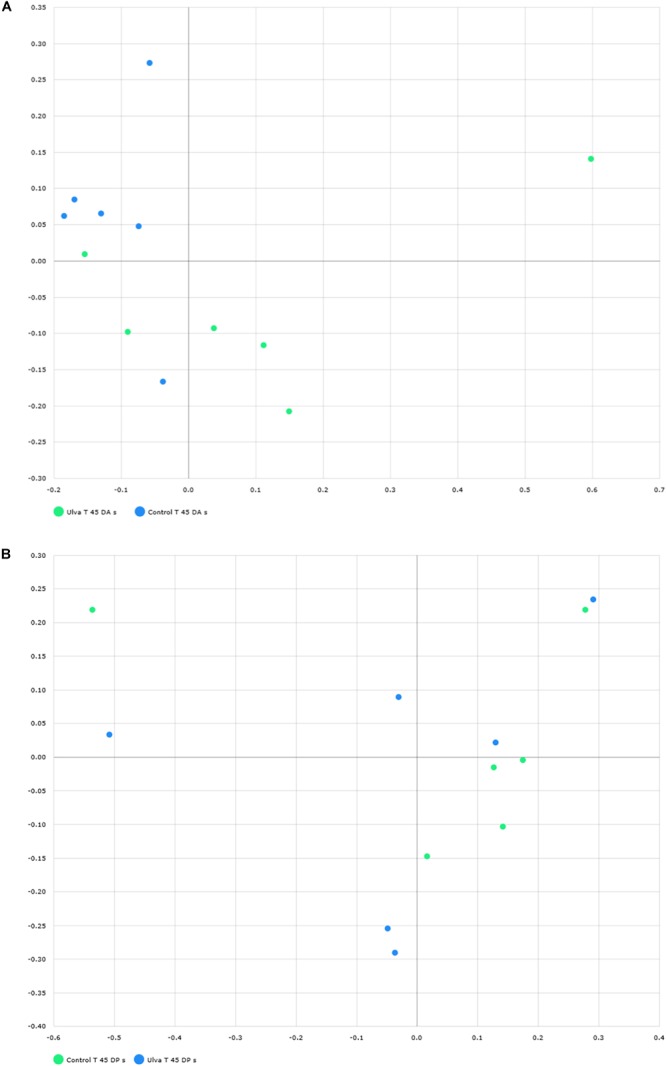
Distribution of GI microbiome samples of *S. senegalensis* specimens fed control and *U. ohnoi* (5%) supplemented diet for 45 days. Principal coordinates analysis was applied based on Bray Curtis index. Microbiota of anterior **(A)** and posterior **(B)** sections in control (green) and *Ulva* (blue) diet groups. **(B)** Microbiota of posterior sections in control (green) and *Ulva* (blue) diet groups.

**FIGURE 4:**
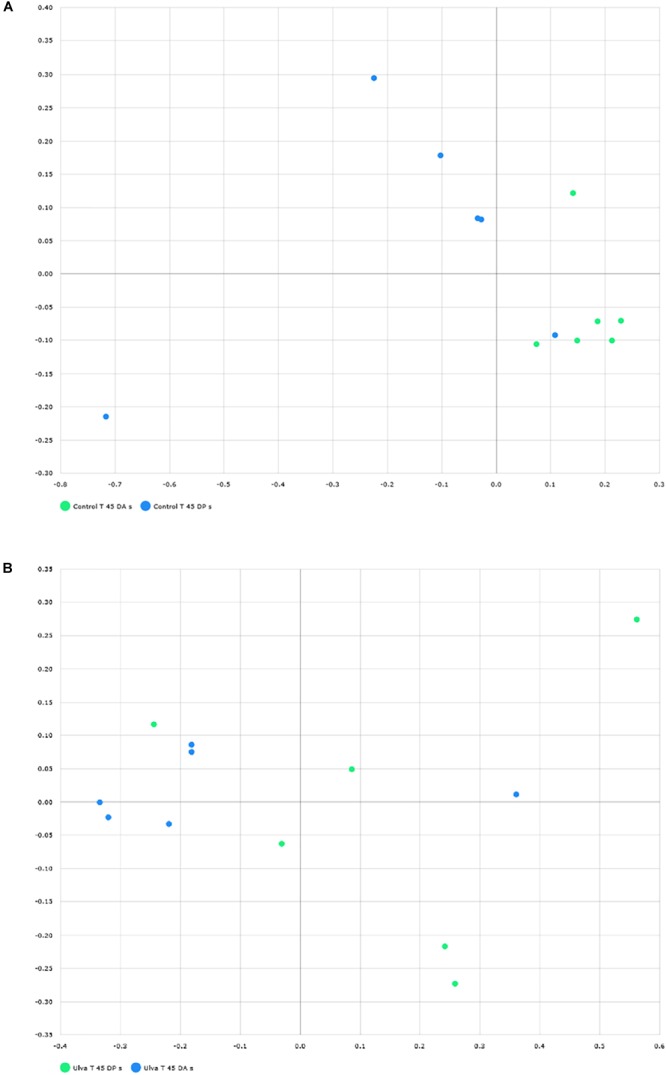
Distribution of GI microbiome samples of *S. senegalensis* specimens fed control and *U. ohnoi* (5%) supplemented diet for 45 days. Principal coordinates analysis was applied based on Bray Curtis index. Microbiota in anterior (blue) and posterior (green) sections offish fed with control diet **(A)** and *Ulva* diet **(B)**.

To study community structure, principal coordinate analysis based on Bray Curtis metric was carried out by using BIOiPLUG comparative MTP analyser. The significance of differences in microbiota composition between samples from fish receiving each diet was tested with permutational multivariate analysis of variance (PERMANOVA) based on Bray-Curtis index. Fish separated in two groups according to the OTUs detected in their anterior GI sections depending on the diet received (Figure 3A) and PERMANOVA analysis revealed an effect of *Ulva* supplementation on the microbiota of the anterior GI tract (*p* = 0.005). However, no separation between fish receiving control and *Ulva* supplemented diet was observed when microbiota of the posterior GI sections was considered (*p* = 0.576) (Figure 3B). Finally, microbiota composition of the anterior and posterior regions was compared for each diet group. In this case, the analysis showed separation of samples based on the GI section in fish fed control diet (*p* = 0.005) but not in the case of those receiving *Ulva* diet (*p* = 0.105) (Figure 4A,B).

In order to determine the OTUs most likely to explain differences between GI sections and diets analyzed, LDA effect size (LEFSe) method according to Segata et al. (2011) was performed. Thus, OTUs differentially abundant between diet groups were detected and effect size values allowed to provide an estimation of the magnitude of the differences between groups due to each OTU detected.

**FIGURE 5:**
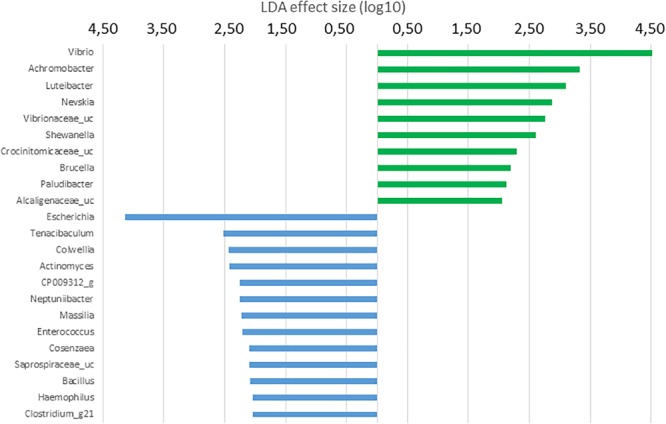
LDA scores for OTUs at genus level differentially abundant in the microbiota of the anterior GI tract of *S. senegalensis* specimens fed control (blue) and *Ulva* supplemented (5%w/w) (green) diets, *p* < 0.05.

**FIGURE 6:**
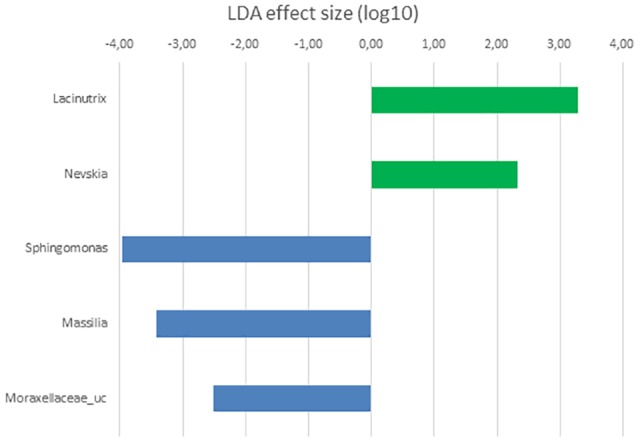
LDA scores for OTUs at genus level differentially abundant in the microbiota of the posterior GI section of *S. senegalensis* specimens fed control (blue) and *Ulva* supplemented (5% w/w) (green) diets, *p* < 0.05.

**FIGURE 7:**
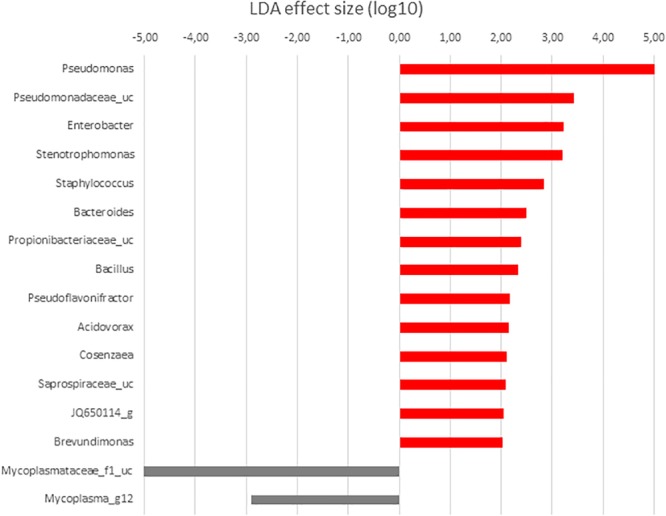
LDA scores for differentially abundant OTUs between anterior (red) and posterior (gray) sections in *S. senegalensis* specimens fed control diet. *p <* 0.05.

**FIGURE 8:**
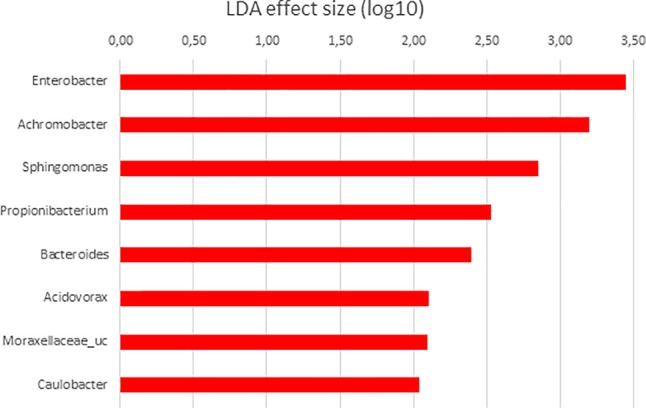
LDA scores for differentially abundant OTUs between anterior (red) and posterior sections in *S. senegalensis* specimens fed with *Ulva* diet. *p <* 0.05.

Histograms of the LDA scores computed for differentially abundant OTUs in the GI microbiota of *S. senegalensis* specimens fed with control and *Ulva* diets and anterior and posterior GI sections are shown in Figure 5–8. LEfSe scores can be interpreted as the degree of consistent difference in relative abundance between features in the two classes of analyzed microbial communities. The histogram identifies the clades that explain the greatest differences between communities. In the case of the anterior GI sections of fish fed with control and *Ulva* diet, the most differentially abundant bacterial taxa (*p* < 0.01) in the microbiota of *Ulva* diet fed specimens belong to Gammaproteobacteria. Several members of *Vibrionaceae* family showed high LDA scores, *Vibrio* genus being the most differentially abundant, with higher abundance percentages in fish receiving dietary *U. ohnoi* (Figure 5). In addition, *Achromobacter* (Betaproteobacteria), *Luteibacter, Nevskia* and *Shewanella* (Gammaproteobacteria) as well as *Crocinitomicaceae_uc* and *Paludibacter* (Bacteroidetes), *Brucella* (Alphaproteobacteria) and *Alcaligenaceae_uc* (Betaproteobacteria) were more abundant in the microbiota of fish fed with the alga.

On the contrary, a wider variety of more differentially abundant taxa was detected in the microbiota of fish fed with control diet. In this case, the most differentially abundant genus was *Escherichia.* Other Gammaproteobacteria including *Colwellia, Neptunibacter, Cosaenzaea*, and *Haemophilus* were also more abundant in the microbiota of fish belonging to the control group. Interestingly, *Tenacibaculum* genus, which includes pathogenic species for *S. senegalensis*, was more abundant in the microbiota of fish fed with the diet devoid of *U. ohnoi*. In addition, *Actinomyces* (Actinobacteria), *Saprospiraceae_uc* (Bacteroidetes) and two members of Firmicutes phylum: *Bacillus* and *Clostridium_g21* were more abundant in the microbiota of control fish.

Differential taxa at genus level in the microbiota of posterior sections of control and *Ulva* supplemented diets fed fish was only represented by *Lacinutrix* (Bacteroidetes) and four Proteobacteria genera: *Nevskia*, more abundant in the microbiota of *Ulva* diet group, and *Sphingomonas, Massilia* and Moraxellaceae_uc, more abundant in the microbiota of control fish (Figure 6). These results are in agreement with the lack of statistically significant differences detected in the previous PERMANOVA analysis of the composition of the microbiota of posterior GI sections.

When the microbiota of anterior and posterior GI sections of fish fed with control diet was compared, highest LDA scores corresponded to Gammaproteobacteria. These taxa were more abundant in anterior sections, a value of 5.24 corresponding to *Pseudomonas* genus (Figure 7), 80.87% relative abundance (Figure 2). Other Gammaproteobacteria such as *Enterobacter, Stenotrophomonas*, and *Cosenzaea* were also more abundant in the anterior GI sections. Firmicutes phylum was also overrepresented in the anterior sections compared to posterior sections. Thus, OTUs identified as *Staphylococcus, Bacillus, Selenomonadaceae*_uc and *Pseudoflavonifractor*, although with low relative percentages, are more abundant in anterior sections. On the contrary, microbiota of posterior sections of control fish was characterized by higher abundance of members of Tenericutes phylum such as *Mycoplasmataceae*_f1 and *Mycoplasma*_g12.

Finally, when microbiota of anterior and posterior GI sections of *Ulva* diet fed specimens were compared, only 8 genera showed LDA scores above 2.00 (Figure 8). However, similar to fish fed control diet, members of Tenericutes were differentially more abundant in the microbiota of posterior sections of fish belonging to the *Ulva* group (Figure 1), though in this case OTUs were identified as Mycoplasmatales_uc (LDA score 3.128, *p* < 0.05). At genus level, differentially abundant taxa were all overrepresented in anterior sections of *S. senegalensis* GI tract. These taxa consisted of *Enterobacter, Achromobacter, Sphingomonas, Acidovorax*, Moraxellaceae_uc and *Caulobacter* (Proteobacteria) as well as *Propionibacterium* (Actinobacteria) and Bacteroides (Bacteroidetes). These data were expected considering the overlapped distribution of samples observed in principal coordinates analysis plots and the lack of differences in the PERMANOVA analysis of microbiota composition (Figure 3, 4).

**FIGURE 9:**
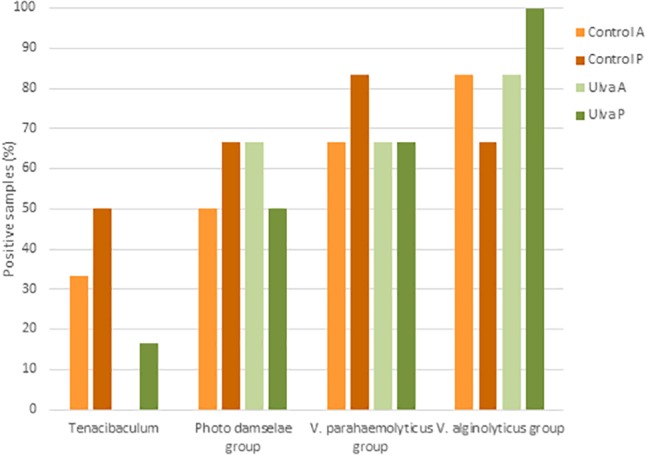
Presence of potential pathogenic taxa in the intestinal microbiota of *S. senegalensis* specimens fed with control or *Ulva* supplemented diet (5% w/w). Values represent percentages of positive samples for *N* = 6 specimens per gastrointestinal section and diet group. A: anterior GI tract sections; P: posterior GI tract sections.

Despite limitations in some closely related species due to the lack of sequence differences, EzBioCloud 16S database can be used for species-level identification in some genera (Kim et al., 2012; Yoon et al., 2017). For this reason, presence of OTUs identified as potential pathogens for *S. senegalensis* including groups such as *Tenacibaculum* spp., *P. damselae* group (including *P. damselae* subsp. *piscicida*), *V. alginolyticus* group (including *V. harveyi)* and *V. parahaemolyticus* group (including *V. parahaemolyticus*) were searched for in the samples analyzed. Presence in the microbiota of anterior and posterior GI sections of specimens fed with each diet assayed is shown in Figure 9. Similar detection percentages of *P. damselae, V. alginolyticus* and *V. parahaemolyticus* groups were observed in the microbiota of Senegalese sole specimens fed with both diets. Although no OTU was identified as *V. harveyi* in the samples studied, presence of members included in *V. alginolyticus* group were detected in samples from fish fed with both control and *Ulva* diet. However, it was not possible to determine whether these OTUs correspond to *V. harveyi* or other species included in *V. alginolyticus* group according to EzBiocloud database.

On the contrary, OTUs identified as *Tenacibaculum* were detected in a lower number of specimens fed with *Ulva* diet (1 out of 6 specimens) compared to control diet (4 out of 6 specimens) and only in the posterior GI section (Figure 9). OTUs belonging to *Tenacibaculum* genus detected in samples from fish fed with control diet were identified as *T. soleae* (1 specimen), *T. litoreum* group including *T. discolor, T. gallaicum, T. litoreum* and *T. ascidiaceicola* (1 specimen), *T. halocynthiae* (3 specimens) and *T. lutimaris* group, including *T. aestuarii* and *T. lutimaris* (3 specimens). OTUs detected in the only positive sample from fish fed with *U. ohnoi* were assigned to *T. lutimaris* group.

## Discussion

### Characterization of *Solea senegalensis* GI Microbiota

A number of studies indicate that diet nature is an important factor in the modulation of the GI tract microbial composition in vertebrates (Askarian et al., 2012; Sullam et al., 2012; Ghanbari et al., 2015; Ringø et al., 2016; Gajardo et al., 2017).

In the present work, NGS techniques (MiSeq) have been used to study *S. senegalensis* GI microbiota for the first time. Overall composition detected in *S. senegalensis* GI microbiota reflects that described for other marine fish species (Verner-Jeffreys et al., 2003; Xing et al., 2013; Egerton et al., 2018; Wang et al., 2018). In the case of *S. senegalensis*, previous studies have reported γ-Proteobacteria as main components of GI microbiota by using DGGE (Tapia-Paniagua et al., 2010, 2014b). At genus level, *Pseudomonas* and *Vibrio* were detected in the microbiota of juvenile *S. senegalensis* specimens (Tapia-Paniagua et al., 2015). Results obtained with NGS techniques in the present work confirm that members of Proteobacteria phylum dominate the microbiota of the GI tract in *S. senegalensis* specimens. Similarly, at genus level, *Pseudomonas* was the most frequently Proteobacteria detected in the present work, especially in anterior sections, although *Vibrio, Acinetobacter*, and *Escherichia* were also present.

*Pseudomonas* species are commonly detected in aquaculture and intestinal environments, it being one of the dominant members of the GI microbiota in marine fish (Wang et al., 2018). In this respect, studies carried out by Tapia-Paniagua et al. (2014a) have detected *Pseudomonas* as a predominant genus in the GI microbiota of *S. senegalensis* larvae (Tapia-Paniagua et al., 2014a), though not in juvenile specimens (Tapia-Paniagua et al., 2014b, 2015). Differences in rearing conditions such as the temperature, with increased abundance of *Pseudomonas* members when water temperature is higher (19°C in the present study, versus 16.6°C) and diet (manufactured diet containing krill and shrimp meal in the present study versus commercial diet), could contribute to differential abundance of *Pseudomonas* in the GI microbiota of Senegalese sole specimens. Nonetheless, this genus was predominant in the intestinal microbiota of juvenile Senegalese sole fed a diet supplemented with a probiotic *Shewanella* strain (Tapia-Paniagua et al., 2015). Changes in the abundance of *Pseudomonas* could be relevant considering that this genus includes species with chitinase, lipase, protease and amylase activities (Ray et al., 2012). Further studies need to be carried out to detect these activities in the species contained in *S. senegalensis* GI tract and verify their role in the digestive process of this fish species.

In this study, *Vibrio* genus was the second most abundant γ-Proteobacteria in *S. senegalensis* GI tract of both fish groups. *Vibrio* has already been reported as a predominant member of the GI microbiota of larvae (Tapia-Paniagua et al., 2015) and juvenile (Tapia-Paniagua et al., 2014a,b, 2015) *S. senegalensis* specimens. In our study, *Vibrio* sequences were mainly assigned to *V. scophthalmi*. This species has been described as the dominant bacterial population in the intestine of healthy reared flat fish such as turbot (*Scophthalmus maximus*) (Cerdá-Cuéllar and Blanch, 2002) and *S. senegalensis* (Tapia-Paniagua et al., 2014b, 2015). In addition, *V. scophthalmi* has been identified as a chitinase-producing bacteria from the digestive tract of Japanese flounder (*Paralichthys olivaceus*) (Sugita and Ito, 2006). However, whether populations of *V. scophthalmi* would allow *S. senegalensis* to use the nutrients derived from chitin needs to be determined. In addition, *V. harveyi* and *V. parahaemolyticus* have been detected as a predominant microorganism in the GI microbiota of *S. senegalensis* specimens farmed under high stocking rearing conditions (Tapia-Paniagua et al., 2014b). Some strains of both species have been reported as opportunistic pathogens for *S. senegalensis* (Zorrilla et al., 2003; Tapia-Paniagua et al., 2015) whereas others were non virulent for this fish (Rico et al., 2008).

Tenericutes was the second most abundant phylum in *S. senegalensis* GI tract. This phylum has previously been detected in the GI tract of both marine and freshwater fish species (Xing et al., 2013; Carda-Dieguez et al., 2014; Givens et al., 2015; Lyons et al., 2017). In the present work, relative abundance of members of this phylum, and its class Mollicutes, mainly phylotype *Mycoplasmataceae*, was differentially higher in the posterior sections of *S. senegalensis* GI tract, where abundance of *Pseudomonas* decreased. *Mycoplasma* has been reported as dominant components in the gut microbiota of carnivorous fish species (Bano et al., 2007; Green et al., 2013) and *S. senegalensis* (Tapia-Paniagua et al., 2014a,b). Composition of the diet used for feeding *S. senegalensis* specimens in the present work included ingredients from animal sources such as fish, squid, krill and shrimp meal, which can have favored *Mycoplasmataceae* populations.

*Mycoplasma* genus includes both pathogenic and saprophytic species. Glucose is the main source of energy for fermentative mycoplasma and a source of carbon for the synthesis of other sugar and carbohydrates. Additionally, acetic and lactic acids are produced by the bacterium (Brown, 2010). *Mycoplasma* species have optimal growth at pH values between 7 and 8 and pH deviations are usually inhibitory for most species (Kirchhoff et al., 1987). *S. senegalensis* GI tract is characterized by a low capacity for acid digestion, with most of the digestion occurring under alkaline conditions (Yúfera and Darias, 2007). This environment would be more favorable for the establishment of *Mycoplasma* populations, explaining the higher abundance of these taxa, especially in the posterior sections of *S. senegalensis* GI tract. As Lyons et al. (2017) suggest for rainbow trout, the dominance of *Mycoplasma* in the posterior sections of *S. senegalensis* GI tract may result from a symbiosis in which the bacterium benefits of the access to host fermentable substrates, and the fish obtains acetic and lactic acid metabolites. In this way, although more research to identify the definite role of *Mycoplasma* is needed, it is interesting to point out that Li et al. (2016) found higher Tenericutes percentages in healthy *Coreius guichenoti* specimens compared to those suffering from furunculosis.

Despite no important changes in pH values are present along *S. senegalensis* GI tract, the anterior and posterior regions differ in their physiological functions, with various sets of digestive enzymes that render different substrates available for microorganisms (Yúfera and Darias, 2007). This situation can explain changes in microbial populations between GI regions indicated above and differences in diversity observed between anterior and posterior sections analyzed in this work. Similar to that reported by Ye et al. (2014) in gizzard shad (*Dorosoma cepedianum*) and Gajardo et al. (2017) in Atlantic salmon, diversity of *S. senegalensis* microbiota was higher in the posterior GI regions. However, other authors have reported no differences between GI regions in Artic charr (*Salvelinus alpinus*) (Nyman et al., 2017) or higher diversity in the anterior locations of the mottled spinefoot rabbitfish (*Siganus fuscescens*) GI tract (Nielsen et al., 2017). These results may reflect the variety of GI tract environments present in fish species and point out to the interest in differentiating along GI locations. In the present work, variations in the microbiota composition along *S. senegalensis* GI microbiota have been described for the first time.

### Effect of Diet Supplementation With Low Percentage of *Ulva ohnoi*

No differences were detected in the richness (Chao1 index) but diversity (Shannon index) in the GI microbiota of *S. senegalensis* increased in fish fed with *Ulva* supplemented diet. These findings may indicate changes in relative abundance distribution of OTU among taxa, but not the presence of new taxa in the microbiota of specimens receiving dietary *U. ohnoi*. Similarly, Givens (2012) and Lyons et al. (2017) reported increased diversity in the microbiota of fish species such as *Lagodon rhomboides* and *O. mykiss*, respectively, fed with diets supplemented with algae. Microbial ecosystem function and stability are influenced by species and functional group richness (Bell et al., 2005), which along with the biodiversity, are essential in protecting ecosystem functionality against changes (De et al., 2014; Goncalves and Gallardo-Escárate, 2017).

In the present work, GI microbiota of *S. senegalensis* specimens fed with the diet containing *U. ohnoi* showed significantly increased abundance of *Vibrio* compared to the microbiota of fish fed the control diet. As no signs or symptoms of disease were observed in these fish during the experimental period, no relationship with disease can be established.

Lower growth and feed conversion rates observed in *S. senegalensis* receiving *Ulva* diet in the present study are in accordance with results in rainbow trout juveniles fed diets with 10% *U. lactuca* reported by Yildirim et al. (2009). The reduced growth observed in fish fed the *Ulva*-supplemented diet could be attributed to the presence of anti-nutritional factors that might be associated with the decreased growth performance. It has been demonstrated that *Ulva*-supplemented diets impaired protein digestibility in salmonids (Pereira et al., 2012; Norambuena et al., 2015) and induced lower protein retention in rainbow trout (Yildirim et al., 2009). Regarding these findings, Sáez et al. (2013) confirmed that *U. rigida* contains substances capable to inhibit the intestinal proteases of seabream juveniles. Indeed, Vizcaíno et al. (2015) reported that intestinal proteolytic activity was reduced after dietary administration of *Ulva* in juvenile seabream in a 70 day-feeding trial.

Thus, in Senegalese sole, the overall lower nutrient gain and growth impairment observed in fish fed *U. ohnoi* might be partially explained by the presence of protease inhibitors that could have adversely affected nutrient uptake through the inhibition of digestive proteases in the intestine. However, Vizcaíno et al. (2015) suggested the existence of a compensation mechanism in seabream owing to that profile of intestinal proteases in fish remains unaffected by dietary inclusion of *Ulva* meal, and no effects on final fish growth was observed. In this sense, microbiota could contribute with its enzymatic pool to compensate this limitation; nonetheless, the effects of *U. ohnoi* on microbial proteases need to be determined. Moreover, Moutinho et al. (2018) reported that dietary inclusion of 10% *U. lactuca* over 5 months (from 23 g up to 60 g) did not have any detectable effect on growth performance and feed utilization in juvenile Senegalese sole. The different response of fish after dietary administration of *Ulva* might be consequence of the different physiological maturation level of specimens used in both feeding trials. Results obtained appointed that early dietary administration of *Ulva* resulted in growth impairment associated with reduced food conversion rate, and for this reason belated administration of *Ulva* could be recommended in the culture of Senegalese sole to avoid the observed effect in the growth when feeding small-size juvenile fish.

On the other hand, microbiota of Senegalese sole specimens fed with the control diet showed differentially higher abundance of OTUs identified as members of *Escherichia* genus. Several authors have reported antibacterial activity against *E. coli* strains in extracts of *Ulva* spp. (Chiheb et al., 2009; Silva et al., 2013). This characteristic could be associated to the lower abundance of this genus in the microbiota of *S. senegalensis* specimens receiving the *Ulva* diet compared to those fed with the control diet.

Presence of pathogens in recirculating aquaculture systems and GI tract of *S. senegalensis* and *S. maximus* specimens without disease symptoms has been previously observed (Martins et al., 2013; Tapia-Paniagua *et al.*, 2014b). *V. parahaemolyticus* strains have been reported as pathogenic for *S. senegalensis* (Zorrilla et al., 2003) and other marine fish species (Abdelaziz et al., 2017). In the present study, sequences identified as *V. parahaemolyticus* and *V. harveyi* groups were detected in the microbiota of fish fed with both diets. Our results are in agreement with the proposal of Givens et al. (2015) who suggested that fish guts might serve as a refuge for *V. parahaemolyticus.* However, results obtained in the present work do not allow us to elucidate if sequences detected correspond to pathogenic species and a carrier status of this fish, or to non-virulent strains.

OTUs assigned to *P. damselae* group including *P. damselae* subsp. *damselae* and *P. damselae* subsp. *piscicida* were also detected in the microbiota of Senegalese sole specimens regardless of the diet received. Previous studies have reported the presence of *P. damselae* subsp. *piscicida* in *S. senegalensis* GI microbiota (Tapia-Paniagua et al., 2014a,b). Similarly, Rico et al. (2016) detected predominant bands related to *Photobacterium* OTUs in DGGE gels when analyzing the GI microbiota of *Sparus aurata* fed diets containing *Ulva rigida* (5%, 15 and 25%). Tapia-Paniagua et al. (2014a,b) and Rico et al. (2016) suggested that the high abundance of naturally antagonistic species in the system may avoid the reach of infective concentrations and would contribute to the control of pathogen populations.

In contrast to previously mentioned pathogens, we have detected significantly lower presence of *Tenacibaculum* in the GI tract of *S. senegalensis* specimens fed with a diet containing *U. ohnoi*. Infections caused by *Tenacibaculum*, affect a wide range of marine species, including Atlantic salmon, flounder (Baxa et al., 1986), turbot (Alsina and Blanch, 1993) seabass (*D. labrax*), and *S. senegalensis* (Avendaño-Herrera et al., 2005). Although the best known and most extensively studied outbreaks are caused by *T. maritimum*, other *Tenacibaculum* spp. are responsible for fish disease (Småge et al., 2016a,b) and species such as *T. discolor* and *T. soleae* have been isolated from diseased Senegalese sole (Piñeiro-Vidal et al., 2008a,b; Morais et al., 2016). Presence of inhibitory compounds against some bacterial species has been reported in *Ulva* extracts (Silva et al., 2013; Reverter et al., 2014). However, this alga does not show high antibacterial activities in their extracts compared to other seaweed species, and inhibition on *Tenacibaculum* spp. has not been reported.

The contribution of the saprophytic microbiota in the control of pathogens has been reported (Kamada et al., 2013). In this study, differentially abundant genera in the GI microbiota of anterior and posterior intestine of *S. senegalensis* receiving *Ulva* diet included genera with strains displaying antagonistic activities such as *Vibrio* (LaPorte, 2017), *Shewanella* (Chabrillón et al., 2005) and *Achromobacter* (Deepa et al., 2015). These results could suggest an important role in the control of *Tenacibaculum* members by microbial populations comprising the microbiota in fish receiving dietary *U. ohnoi*. Furthermore, the detection of pathogenic species in Senegalese sole specimens jointly with the lack of signs of disease could indicate the presence of a carrier stage and the control of pathogen populations. In this way, the presence of *T. maritimum* in the intestinal tract of apparently healthy fish has been reported by some authors, suggesting that the intestine may act as a reservoir for this pathogen (Faílde et al., 2013). Results obtained in the present work indicate that a diet containing *U. ohnoi* (5%) is associated to lower presence of *Tenacibaculum* in the GI tract, thus it being able to limit not only the risk of pathology in the fish but also the potential transmission of the pathogen to other hosts.

## Conclusion

Differences in the microbiota composition of anterior and posterior sections of *S. senegalensis* GI tract have been observed, *Pseudomonas* being more abundant in the anterior sections and *Mycoplasmataceae* the dominant taxon in the posterior GI tract sections. On the other hand, modulation of the GI microbiota of juvenile Senegalese sole fed with a diet containing low percentage of *U. ohnoi* for 45 days has been detected in the present study. Microbiota of anterior regions of GI tract has been mainly been modulated and a significant reduction in the presence of *Tenacibaculum* has been observed in fish specimens receiving dietary *U. ohnoi*.

## Author Contributions

ST-P and MF performed the sampling, DNA extraction, and data collection. CF-D and VA participated in sampling, cultivation of the algae, and fish maintenance. FA prepared the aquafeed. MM and MB performed the data analysis and wrote the manuscript. MB, MM, CF-D, and FA designed the work. All authors contributed to the discussion and final writing.

## Conflict of Interest Statement

The authors declare that the research was conducted in the absence of any commercial or financial relationships that could be construed as a potential conflict of interest.

## Footnotes

1 http://www.ual.es/stecnicos_spe
